# Concepts of neural nitric oxide-mediated transmission

**DOI:** 10.1111/j.1460-9568.2008.06285.x

**Published:** 2008-06

**Authors:** John Garthwaite

**Affiliations:** Wolfson Institute for Biomedical Research, University College LondonGower Street, London WCIE 6BT, UK

**Keywords:** cGMP, guanylyl cyclase, retrograde messenger, synaptic plasticity

## Abstract

As a chemical transmitter in the mammalian central nervous system, nitric oxide (NO) is still thought a bit of an oddity, yet this role extends back to the beginnings of the evolution of the nervous system, predating many of the more familiar neurotransmitters. During the 20 years since it became known, evidence has accumulated for NO subserving an increasing number of functions in the mammalian central nervous system, as anticipated from the wide distribution of its synthetic and signal transduction machinery within it. This review attempts to probe beneath those functions and consider the cellular and molecular mechanisms through which NO evokes short- and long-term modifications in neural performance. With any transmitter, understanding its receptors is vital for decoding the language of communication. The receptor proteins specialised to detect NO are coupled to cGMP formation and provide an astonishing degree of amplification of even brief, low amplitude NO signals. Emphasis is given to the diverse ways in which NO receptor activation initiates changes in neuronal excitability and synaptic strength by acting at pre- and/or postsynaptic locations. Signalling to non-neuronal cells and an unexpected line of communication between endothelial cells and brain cells are also covered. Viewed from a mechanistic perspective, NO conforms to many of the rules governing more conventional neurotransmission, particularly of the metabotropic type, but stands out as being more economical and versatile, attributes that presumably account for its spectacular evolutionary success.

## Introduction

Research into nitric oxide (NO) signalling in the nervous system continues to be fascinating and challenging. It emerged as a signalling molecule in the brain 20 years ago following the search for a missing transmitter that was generated in response to neuronal NMDA receptor activation and caused cGMP generation in other cells (Table 1). Since that time, NO has been implicated in many different neural functions. In peripheral organs, including those of the digestive, respiratory and urogenital tracts, NO performs a neurotransmitter-like role, being released from nitrergic nerves to mediate smooth muscle relaxation (reviewed in Rand & Li, 1995; Toda & Okamura, 2003; Toda & Herman, 2005). In the vertebrate central nervous system (CNS), NO is associated with many different behaviours, including learning and memory formation, feeding, sleeping and male and female reproductive behaviour, as well as in sensory and motor function. Some of these broad roles have been conserved through millions of years of evolution, in some cases dating back to animals with the most primitive nervous systems. In a type of jellyfish, for example, NO evokes the rhythmic swimming pattern associated with feeding, apparently by binding to receptors coupled to cGMP formation, much like the receptors operating in mammals (Moroz *et al.*, 2004). From some of these early cnidarians through to insects and molluscs, a principal function of NO is to regulate olfaction and feeding and their related learning behaviours (see reviews by Davies, 2000; Moroz, 2001; Trimmer *et al.*, 2004; Palumbo, 2005). Following this trend through into mammals, common phenotypes of mice deficient in either neuronal NO formation (Huang *et al.*, 1993), or NO receptors (Friebe *et al.*, 2007) or a downstream protein kinase enzyme (Pfeifer *et al.*, 1998), are disturbances in gastrointestinal function.

**Table 1:** Key papers leading to the identification of NO as a brain transmitter

Reference	Main findings
Ferrendelli *et al.* (1974, 1976)	Glutamate elicits Ca^2+^-dependent elevation of cGMP in brain slices; speculated on an intervening transmitter
Arnold *et al.* (1977) and Miki *et al.* (1977)	NO activates soluble guanylyl cyclase in brain homogenates
Deguchi & Yoshioka (1982)	l-arginine identified as the endogenous activator of soluble guanylyl cyclase in brain extracts; activation similar to that induced by NO-releasing agents
Garthwaite (1985)	cGMP response to glutamate in dispersed brain cells mediated exclusively through NMDA receptors
Garthwaite & Garthwaite (1987)	cGMP response to NMDA involves cell-cell communication; missing transmitter presumed unstable and could be substituted by exogenous NO (from sodium nitroprusside)
Palmer *et al.* (1987) and Ignarro *et al.* (1987)	Endothelium-derived relaxing factor (EDRF) in blood vessels identified as NO
Garthwaite *et al.* (1988)	Brain transmitter identified as EDRF/NO; released Ca^2+^-dependently on NMDA receptor stimulation

There have been a large number of reviews during the last few years on the roles of NO in many aspects of vertebrate CNS function, including neurogenesis, neuronal differentiation and development (Mize & Lo, 2000; Contestabile & Ciani, 2004; Estrada & Murillo-Carretero, 2005), memory and other behaviours (Golombek *et al.*, 2004; Susswein *et al.*, 2004; Argiolas & Melis, 2005; Del Bel *et al.*, 2005; Nelson *et al.*, 2006), and neuropathology and/or neuroprotection (Dawson & Dawson, 1998; Contestabile *et al.*, 2003; Keynes & Garthwaite, 2004; Duncan & Heales, 2005; Blokland *et al.*, 2006; Zhang *et al.*, 2006a; Calabrese *et al.*, 2007). The present article attempts to step inside the broader physiological roles of NO and, taking advantage of the substantial recent progress in understanding the cellular and molecular mechanisms it engages, to start to build a picture of how it operates as a signalling molecule.

## NO synthesis

Much is known about the mechanism of NO synthesis from biochemical studies of the purified NO synthase (NOS) enzymes. They are complex proteins found constitutively in two isoforms, neuronal (nNOS) and endothelial (eNOS). The third, inducible, type (iNOS) is rarely present normally but can be expressed in numerous cell types (prototypically in macrophages, mainly in microglia in the CNS) when subjected to immunological challenge. All three isoforms generate NO from l-arginine but have distinct functional and structural features (reviewed by Alderton *et al.*, 2001; Stuehr *et al.*, 2004). iNOS is usually linked with pathological situations and will not be considered here.

### nNOS

The first NO synthase to be purified and cloned (Bredt & Snyder, 1990; Bredt *et al.*, 1991b) and the most abundant isoform generally found in the central and peripheral nervous systems is nNOSα, which is activated by Ca^2+^ complexed with calmodulin and has a wide but uneven distribution in the mammalian brain, resembling in extent that of a major neurotransmitter (Bredt *et al.*, 1991a; Vincent & Kimura, 1992). The physical association of nNOSα and the NMDA receptor subunit NR2B with postsynaptic density protein-95 (PSD-95) through their specialised PDZ domains (Brenman *et al.*, 1996) helps explain the preferential link between NMDA receptors and NO production (Garthwaite, 1985; Garthwaite *et al.*, 1988). Other splice variants also exist, namely nNOSβ and nNOSγ, both of which lack the amino terminal PDZ domain (Brenman *et al.*, 1996). nNOSγ has little or no enzymatic activity but nNOSβ is active and is upregulated in the striatum and cortex in mice lacking the nNOSα isoform (Eliasson *et al.*, 1997; Langnaese *et al.*, 2007), which probably accounts for the relatively mild phenotype of such animals compared to ones lacking the β and γ variants as well (Gyurko *et al.*, 2002).

In addition to regulation by Ca^2+^/calmodulin, nNOS possesses several putative sites for phosphorylation. Phosphorylation by cAMP- and cGMP-dependent protein kinases, by protein kinase C and by Ca^2+^/calmodulin-dependent protein kinase (CaMK)II were reported for the purified enzyme early on, but the effects on activity were generally quite modest and sometimes contradictory (Nakane *et al.*, 1991; Bredt *et al.*, 1992; Dinerman *et al.*, 1994b). A resurgence of interest in this type of post-translational regulation has come from research carried out on cells. CaMKII, a co-resident with NMDA receptors and nNOS at synapses (Kennedy, 2000), was found to phosphorylate the enzyme on serine-847 and inhibit NO formation by ∼50%, probably by affecting Ca^2+^/calmodulin binding (Hayashi *et al.*, 1999; Komeima *et al.*, 2000). When studied in cultured hippocampal neurones, glutamate had a dual effect on nNOS phosphorylation on serine-847, increasing it at low concentrations (5 μm) and decreasing it at the high concentrations more usually associated with excitotoxicity (100 μm or more). Both were mediated by NMDA receptors, with the phosphorylation blocked by CaMKII inhibition and dephosphorylation by concentrations of okadaic acid active on protein phosphatase 1 (Rameau *et al.*, 2004). nNOS phosphorylated on serine-847 was found to exist in rat brain (Hayashi *et al.*, 1999), indicating it to be a physiologically relevant modification. In the cultured neurones, phosphorylated nNOS was concentrated in dendritic spines but the phosphorylation process was slow, taking 15 min to be detectable (Rameau *et al.*, 2004), suggesting that CaMKII is likely to be not a dynamic regulator of nNOS activity but more a longer-term gain controller.

The protein kinase Akt (also known as protein kinase B) phosphorylated nNOS in cultured cortical neurones on serine-1412 (Rameau *et al.*, 2007). This site is equivalent to a key one in eNOS (see below) and its phosphorylation was evoked by 5 min exposure to low glutamate concentrations (5–30 μm) or by briefer periods (1 min) at higher concentrations, after which dephosphorylation became overwhelming. Serine-1412 phosphorylation was NMDA receptor-dependent and led to a rapid enhancement of NOS activity (by how much remains unclear), with dephosphorylation being dependent on AMPA receptors and L-type Ca^2+^channels. Blockers of either of these activities also enhanced serine-1412 phosphorylation in the absence of glutamate, suggesting that Ca^2+^influx through L-type channels in response to AMPA receptor-mediated depolarization tonically stimulates phosphatases that regulate NMDA receptor-associated nNOS activity. The presence of serine-1412-phosphorylated nNOS in a rat brain lysate (Rameau *et al.*, 2007) indicates that the modification occurs *in vivo* and it may produce a stimulatory effect (as with eNOS; see below) by increasing the sensitivity of nNOS to Ca^2+^/calmodulin (Adak *et al.*, 2001; Rameau *et al.*, 2007).

Phosphorylation of nNOS in neuroblastoma cells incubated with a phosphatase inhibitor occurred on threonine-1296 resulting in an ∼50% reduction in activity although a mutation mimicking threonine-1296 phosphorylation showed a stronger effect (Song *et al.*, 2005). CaMKI may inhibit nNOS activity though serine-741 (Song *et al.*, 2004). No evidence yet exists for phosphorylation of either of these sites *in vivo*.

In addition to phosphorylation, nNOS activity or its location may be influenced by interactions with a number of proteins (Rodriguez-Crespo *et al.*, 1998; Billecke *et al.*, 2002; Jaffrey *et al.*, 2002; Dreyer *et al.*, 2004) but additional work is needed to clarify their functional significance. Recent evidence indicates that nNOS can bind to the serotonin transporter in the plasma membrane such that serotonin uptake then couples to NO formation (Chanrion *et al.*, 2007), a hitherto unique ‘metabotropic’ transporter activity (Garthwaite, 2007).

These new findings suggest that the regulation of nNOS activity in neurones is more complex than previously thought and suggest intriguing options for cross-talk with other signalling pathways. Phosphorylation may transpire to be at least as important for nNOS as it is for eNOS.

### eNOS

Based on immunocytochemical staining, this isoform was originally claimed to be present in neurones of the hippocampus (Dinerman *et al.*, 1994a; O’Dell *et al.*, 1994) but later studies inferred that the staining was artifactual (Demas *et al.*, 1999; Blackshaw *et al.*, 2003). Its presence or otherwise in astrocytes remains unsettled, some finding by immunocytochemistry or *in situ* hybridisation that only endothelial cells in the brain were labelled (Seidel *et al.*, 1997; Stanarius *et al.*, 1997; Topel *et al.*, 1998; Demas *et al.*, 1999; Blackshaw *et al.*, 2003) whereas others reported immunocytochemical evidence for astrocytic eNOS (see the recent review of the literature by Lin *et al.*, 2007). Tests with eNOS-knockout mice would help determine whether the astrocyte staining is specific or not. In the meantime, endothelial cells are probably the major, if not the sole, location of eNOS in brain tissue. Endothelial eNOS is of emerging relevance in the regulation of brain function independently of its role in the vasculature (see below) and it continues to be the subject of much research, not least because of its importance in cardiovascular function and malfunction.

The controls over eNOS activity are multifarious (reviewed in Cirino *et al.*, 2003; Dudzinski *et al.*, 2006). Catalytically active eNOS in endothelial cells is largely tied to the plasma membrane by a lipid modification (palmitoylation) and resides in specialised invaginations (caveoli) in association with other proteins, including caveolin-1 and heat-shock protein (Hsp)-90. Dissociation from caveolin-1 is required for activity and is promoted by Ca^2+^/calmodulin binding. Despite this, eNOS is often tonically active in blood vessels, perhaps the most important mechanism for sustaining the activity being phosphorylation on serine-1179, enabling the enzyme to function at resting cytosolic Ca^2+^ concentrations. The prototype kinase mediating serine-1179 phosphorylation is Akt, which is activated by phosphoinositide-3 kinase in response to stimuli such as shear stress, oestrogens, insulin and vascular endothelial growth factor, but the same site is also targeted by AMP kinase (in response to metabolic stress), protein kinase C, cAMP- and cGMP-dependent protein kinases, and CaMKII.

## NO receptors

The activation by NO of ‘soluble’ guanylyl cyclase in homogenates of various tissues was discovered well in advance of any inkling that NO was of biological relevance (Arnold *et al.*, 1977; Miki *et al.*, 1977), and proved vital to the hypothesis that the endothelium-derived relaxing factor was NO (Furchgott, 1999; Ignarro, 1999). It remains the only recognized physiological NO signal transduction mechanism and much evidence has accrued during the last several years confirming its pre-eminence in transducing the actions of endogenous NO (Krumenacker *et al.*, 2004), the most striking example of which being the complete loss of NO-mediated vascular relaxation following genetic deletion of NO-activated guanylyl cyclases (Friebe *et al.*, 2007). The homogenate-based name (soluble guanylyl cyclase) is still widely used but it has little meaning in a cellular context, and was never meant to (Chrisman *et al.*, 1975). In reality, the proteins are enzyme-linked receptors that, in cells, are often associated indirectly with membranes (see below) and here they are simply called ‘NO receptors’.

In common with all other receptors, NO receptors are equipped with a ligand binding site and a transduction domain but in many ways they are especially fascinating. The ligand binding site is an unremarkable haem group of the type used in haemoglobin for binding O_2_ but, when incorporated into the receptor protein, it exhibits amazing preference for NO, allowing cellular NO signalling to occur in the presence of > 10 000-fold excess of O_2_, despite the close chemical similarity of the two ligands (Stone & Marletta, 1994; Martin *et al.*, 2006). NO detection by protein-associated haems appears to have evolved in certain bacteria and to have been incorporated into eukaryotic animals by horizontal gene transfer (Iyer *et al.*, 2003; Fitzpatrick *et al.*, 2006). The protein component is an αβ-heterodimer of which there are two known isoforms, α1β1 and α2β1. They comprise a haem-binding region, a dimerisation domain and a catalytic domain where GTP is converted to cGMP. The catalytic domain is very similar to that of adenylyl cyclase, to the extent that switching three amino acids converts NO-activated guanylyl cyclase into NO-activated adenylyl cyclase (Sunahara *et al.*, 1998). In the inactive state, the ligand-binding haem group is coordinated to the protein by a histidine bond (Wedel *et al.*, 1994; Zhao *et al.*, 1998) and, by analogy with adenylyl cyclase, the catalytic domain in the inactive state is in an open configuration (Dessauer *et al.*, 1999). Binding of NO to the haem is almost diffusion-limited (Makino *et al.*, 1999; Zhao *et al.*, 1999) and, from structural studies on a homologous cyanobacterial NO sensor (Ma *et al.*, 2007), this event causes the haem to pivot which, together with a dislocation of the coordinating histidine group, results in a conformational change in the protein that propagates to the catalytic domain, resulting in domain closure and catalysis.

When studied in a cell-free environment, the α1β1 and α2β1 NO receptor isoforms are similarly sensitive to NO, half-maximal activity being evoked at a concentration of ∼1 nm (Wykes & Garthwaite, 2004). Other properties, such as the maximal guanylyl cyclase activity and pharmacological properties, also appear very similar (Russwurm *et al.*, 1998; Gibb *et al.*, 2003) but whether this pertains to the proteins in their natural environment is not known. The potency of NO for its receptors in cells (EC_50_ = 10 nm) is an order of magnitude lower than that of the purified protein (Mo *et al.*, 2004; Roy & Garthwaite, 2006), which is partly explained by the presence in cells of ATP (which, although inhibitory, accelerates the kinetics) and a lower GTP concentration than is typically used in biochemical assays (B. Roy, E.J. Halvey and J. Garthwaite, unpublished observations). In brain cells, NO switches on the associated guanylyl cyclase activity with no observable delay (with a 20-ms sampling time) and, on removal of NO, the activity decays with a half-time of 200 ms (Bellamy & Garthwaite, 2001b), kinetics not dissimilar to that of NMDA receptors or of metabotropic GABA or glutamate receptors (Dale & Roberts, 1985; Dutar & Nicoll, 1988; Batchelor & Garthwaite, 1997).

Although widespread, the two NO receptor isoforms have a differing cellular distribution in the brain (Budworth *et al.*, 1999; Gibb & Garthwaite, 2001; Mergia *et al.*, 2003; Szabadits *et al.*, 2007) and, at the subcellular level, may also have different locations because the α2β1 receptor binds through its PDZ domain to proteins that are enriched in synapses, namely PSD-95 and putatively also to the related proteins PSD-93, SAP-97 and SAP-102 (Russwurm *et al.*, 2001). The other isoform, α1β1, may be cytosolic in part but there is evidence that it too may become plasma membrane-associated under conditions of raised intracellular Ca^2+^ (Zabel *et al.*, 2002) or exposure to cannabinoids (Jones *et al.*, 2008). Membrane association may depend on binding to Hsp-90 (Venema *et al.*, 2003; Papapetropoulos *et al.*, 2005; Nedvetsky *et al.*, 2008), Hsp-70 (Balashova *et al.*, 2005) or other proteins (Meurer *et al.*, 2004). In homogenates, varying proportions of total NO-activated guanylyl cyclase activity are found in membrane fractions (Arnold *et al.*, 1977): for example, in rat platelets, this component amounts to 60% of the activity in the cytosol whereas, in the cerebellum, it is half this amount (Wykes & Garthwaite, 2004). In homogenates of platelets and cerebellum, NO was equipotent (EC_50_∼1 nm) on the receptors in supernatant and membranes (Wykes & Garthwaite, 2004). The biological significance of the shuttling of α1β1 NO receptors to the membrane (and, presumably, back again) is unknown. The linkage between α2β1 receptors and membrane-associated synaptic proteins may position them within close range of the sites of NO release, which would be particularly important if NO signalling operates in discrete spatial dimensions (see below). Conceivably, by placing the receptor closer to an NO source, attachment of the α1β1 isoform to the outer cell membrane could perform a similar function. In subcellular dimensions, this positioning could make the difference between the receptors being accessible to NO generated in a nearby cellular compartment and being out of range (see below).

As judged by immunocytochemistry of NO receptor proteins (Ding *et al.*, 2004) and of cGMP following exposure to NO sources (Southam & Garthwaite, 1993; de Vente *et al.*, 1998), NO receptors are present to a greater or lesser extent throughout the CNS and have a distribution complementary to that of nNOS, consistent with the two being functional partners. A growing number of good pharmacological tools targeting different sites are now available for manipulating NO–cGMP signalling at the receptor level (Table 2). NO receptors are potential substrates for phosphorylation by several kinases but physiological regulation through such modifications remains to be clarified (reviewed in Pyriochou & Papapetropoulos, 2005). Recent evidence indicates that phosphorylation by cAMP-dependent protein kinase enhances the associated guanylyl cyclase activity at resting levels of NO in pituitary cells (Kostic *et al.*, 2004) whereas, in gastrointestinal smooth muscle cells, activation of muscarinic M2 receptors reduces cGMP generation through Src kinase-dependent tyrosine phosphorylation of the receptor (Murthy, 2008).

**Table 2:** Pharmacology of NO receptors

Class of agent	Selected compounds (original paper)	Comments
Inhibitors of ligand binding	ODQ (Garthwaite *et al.*, 1995) and NS 2028 (Olesen *et al.*, 1998)	Act by oxidising the haem iron, thereby inhibiting NO binding (Schrammel *et al.*,1996); oxidation may predispose the receptors to haem loss and then protein degradation (Stasch *et al.*, 2006).
Allosteric enhancers	YC-1 (Ko *et al.*, 1994) and BAY 41-2272 (Stasch *et al.*, 2001)	Potentiate NO-evoked activity by slowing the rate of deactivation (Friebe & Koesling, 1998). YC-1 also affects cGMP hydrolysing phosphodiesterases at similar concentrations to those active on the receptor (Galle *et al.*, 1999), whereas BAY 41-2272 is more selective (but see Bischoff & Stasch, 2004; Mullershausen *et al.*, 2004).
Ligand binding site (haem) mimetics	Protoporphyrin IX (Ignarro *et al.*, 1982), BAY 58-2667 (Stasch *et al.*, 2002) and HMR1766, S3448 (Schindler *et al.*, 2006)	Activate the haem-free species (Schmidt *et al.*, 2004; Roy *et al.*, 2008) which normally appears to be a very small proportion of the total but which can nevertheless evoke functional responses when engaged pharmacologically. Protoporphyrin IX is a partial agonist and zinc protoporphyrin IX an antagonist for this site; the haem-free receptor targeted by the compounds may become more abundant in disease states (Stasch *et al.*, 2006).

### Transduction of cGMP signals

Direct actions of cGMP can be exerted by binding to agonist or regulatory sites on cyclic nucleotide-gated (CNG) ion channels (reviewed in Kaupp & Seifert, 2002) or hyperpolarization-activated, cyclic nucleotide-modulated (HCN) channels (reviewed in Craven & Zagotta, 2006). cGMP also binds directly to the phosphodiesterase (PDE) enzymes PDE2, PDE5 and, in retinal photoreceptor cells, PDE6, resulting in heightened catalytic activity and cGMP breakdown. cGMP is a low-efficacy substrate for another PDE, PDE3, so that cGMP binding to the catalytic site leads to inhibition of cAMP hydrolysis, potentially raising cAMP levels (reviewed by Bender & Beavo, 2006). Probably the most widespread mechanism employed by cGMP is activation of PKG, which exists in three forms, PKG1α and PKG1β (splice variants) and PKGII, which is anchored to the plasma membrane by myristoylation. PKG1α is concentrated in cerebellum and dorsal root ganglia and PKG1β in the hippocampus and olfactory bulb, whereas PKGII has a more widespread distribution in the brain but shows a particular abundance in the thalamus (reviewed in Feil *et al.*, 2005; Vaandrager *et al.*, 2005; Hofmann *et al.*, 2006). Several substrates for PKG have been identified (reviewed in Schlossmann & Hofmann, 2005) and many of its actions are exerted at the level of phosphatases, leading indirectly to increased or decreased levels of phosphorylation of effector proteins.

Whilst traditionally acting intracellularly, cGMP is also found extracellularly in the brain, where its levels fluctuate according to changes in endogenous NO formation (reviewed by Vincent *et al.*, 1998; Pepicelli *et al.*, 2004). cGMP can be exported from cells through members of the multidrug resistance protein family (reviewed by Sager, 2004) and might serve an additional intercellular signalling role, consistent with evidence that extracellularly applied cGMP has biological effects (Touyz *et al.*, 1997; Poulopoulou & Nowak, 1998; Montoliu *et al.*, 1999).

### Hydrolysis of cGMP

Most of the 11 known PDE families can hydrolyse cGMP, those with the highest affinity being PDE1, 2, 3, 5, 6, 9, 10 and 11 (see review by Bender & Beavo, 2006). Little is known about the PDE isoforms responsible for cGMP hydrolysis in individual cell types, and there may be mixtures at work. For example, cerebellar Purkinje cells all appear to contain PDE5 but a subset additionally expresses a PDE1 isoform (Shimizu-Albergine *et al.*, 2003); in cerebellar astrocytes PDE5 is also prominent but PDE4, which has low affinity (> 100 μm) for cGMP (∼1 μm for cAMP), also contributes when cGMP reaches high levels (Bellamy & Garthwaite, 2001a); in ventrobasal thalamic neurones, on the other hand, PDE2 and putatively PDE9 appear most important (Hepp *et al.*, 2007); PDE2 also plays a major role in hydrolyzing NO-evoked increases in cGMP levels in hippocampus and striatum (van Staveren *et al.*, 2001; Wykes *et al.*, 2002; Boess *et al.*, 2004) whereas, in pituitary nerve terminals, PDE5 is again significant (Zhang *et al.*, 2007b). Suitably selective inhibitors, now available for a number of PDEs, give a complementary strategy for investigating NO receptor signalling at various levels, including in memory performance *in vivo* (reviewed by Blokland *et al.*, 2006).

## Other transduction pathways

There are some examples of physiological NO signals being transduced in a cGMP-independent manner in the nervous system (Jacoby *et al.*, 2001; Lev-Ram *et al.*, 2002), implying the existence of other NO receptors yet to be identified.

One chemical process that has been hailed by some as a physiological NO signal transducing mechanism is the nitrosation of protein thiol groups (see Stamler *et al.*, 2001), or *S*-nitrosation (often confusingly called *S*-nitrosylation; see discussion by Koppenol, 2002), but this idea remains highly controversial. It is facile to evoke protein *S*-nitrosation by exposing cells to high concentrations of nitrosothiols (e.g. *S*-nitroso-*N*-acetylpenicillamine) which can transfer their NO moiety onto other thiols, or to concentrations of exogenous NO that produce nitrosating species on reaction with oxygen (e.g. N_2_O_3_), or by unphysiologically exposing cells to a Ca^2+^ ionophore (reviewed by Hogg, 2002), but there are no unambiguous instances of neural function being regulated physiologically through this modification. *S*-nitrosation of synaptic NMDA receptors was once advertised as a negative feedback mechanism (Lipton *et al.*, 2002) but, on experimental testing, was found to be an artifact (Hopper *et al.*, 2004). Working with NO is undoubtedly difficult and experiments are susceptible to various artifacts arising from unintended chemical and biological reactions (reviewed in Keynes & Garthwaite, 2004). Even seemingly innocuous ingredients, such as Hepes buffer, and conducting experiments using tissue culture media under normal laboratory lighting conditions, can cause problems (Beckman & Koppenol, 1996; Keynes *et al.*, 2003). *In vivo*, *S*-nitrosation may occur mainly in pathological states where the changed redox environment may facilitate the appearance of species capable of converting thiols to nitrosothiols (see review by Zhang & Hogg, 2005).

Another speculative physiological target for NO is mitochondrial cytochrome *c* oxidase (Erusalimsky & Moncada, 2007), which reduces O_2_ to water. NO can compete with O_2_ for binding to this complex and thereby inhibit respiration but higher concentrations are needed than to activate receptors, the EC_50_ value at physiological O_2_ concentrations (20–30 μm) being 120 nm (Bellamy *et al.*, 2002). In an *in vivo* investigation, cytochrome *c* oxidase in brain was unaffected by inhibition of NO production either before or after a period of ischaemia (De Visscher *et al.*, 2002) and, in *in vitro* studies on brain slices, the prevailing NO concentrations appeared to remain in the low nanomolar range or below (too low to affect mitochondrial respiration) despite intense NMDA receptor activation (Bellamy *et al.*, 2002; Keynes *et al.*, 2004), transient simulated ischaemia (Griffiths *et al.*, 2002), or the expression of active iNOS in microglia (Duport & Garthwaite, 2005). Only by artificially increasing the numbers of activated microglia in hippocampal slice cultures could NO receptors be seen to be saturated, suggesting ambient concentrations in excess of 10 nm (Duport & Garthwaite, 2005). Hence, mitochondrial inhibition by NO in the brain may only become relevant under certain pathological conditions, for example when iNOS is expressed in active inflammatory plaques in multiple sclerosis (reviewed in Smith & Lassmann, 2002).

## NO inactivation

It is often stated that NO does not need a specialised inactivation mechanism because it is disposed of by virtue of its natural reactivity. However, at the low nanomolar concentrations and below that are likely to be physiological, NO is remarkably unreactive. Reaction with O_2_, for example, is obvious at micromolar NO concentrations but negligible at low nanomolar concentrations (Ford *et al.*, 1993). Reaction with superoxide ions, giving peroxynitrite, is extremely rapid (Nauser & Koppenol, 2002) but, over the presumed physiological NO concentration range, superoxide dismutase is greatly in excess of NO so that superoxide ions are removed too quickly to allow reaction with NO (Beckman & Koppenol, 1996). Low NO concentrations will, however, react avidly with lipid peroxyl radicals in a beneficial process that stops lipid peroxidation (O’Donnell *et al.*, 1997; Keynes *et al.*, 2005). In physiological conditions, one pathway for NO degradation will be through reaction with haemoglobin in circulating erythrocytes, forming nitrate and methaemoglobin (Liu *et al.*, 1998). Calculations based on the geometry of the microcirculation suggest that this pathway would impose on NO a tissue half-life of ∼1 s (J. Wood and J. Garthwaite, unpublished result).

A much more active mechanism for NO consumption exists in brain tissue itself. Reminiscent of the effect of transporters on the potency of exogenous glutamate (Garthwaite, 1985), it was found that almost 1000-fold higher NO concentrations were required to saturate NO receptors in incubated slices of cerebellum than in dispersed cells (where diffusional barriers are lacking), implying rapid consumption of NO as it diffuses into the slices (Hall & Garthwaite, 2006). At a gross level, consumption appeared to be uniform across the slice, suggesting that the mechanism is present in all cerebellar cell layers. Moreover, NO consumption could not be detected in blood platelets and leukocytes (Keynes *et al.*, 2005), and appears to be very weak in the intact aorta (Liu *et al.*, 2008), pointing to a specialised mechanism and, parenthetically, also explaining why exogenous NO is so much less potent at activating receptors in cerebellar tissue than in blood vessels (Southam & Garthwaite, 1991). From a diffusion-inactivation model, the mechanism in the cerebellar slices conformed to a Michaelis–Menten-type reaction having a maximum velocity of 1–2 μm/s and a Michaelis constant of ∼10 nm. From these values, it is predicted that inactivation would impose a very short half-life (< 10 ms) on NO in concentrations up to 10 nm (Hall & Garthwaite, 2006). This process would have little effect on single sources of NO, where diffusional dispersion would dominate, but would impinge strongly where there are multiple sources within a tissue volume, such as when a plexus of nerve fibres are active (Philippides *et al.*, 2005) or when NO is generated in the microvascular network. The mechanism could not be explained by any known method of NO consumption and it remains to be identified. It is worth noting, however, that its activity may vary from one brain region to another: in slices containing the nucleus of the solitary tract (Wang *et al.*, 2007), exogenous NO affected neurotransmission at concentrations (∼1 nm) that would be expected to be active without any tissue NO consumption. Because of the steep NO concentration gradients across the slice thickness imposed by NO consumption (Hall & Garthwaite, 2006), the location of the cell under study relative to the slice surface would also dictate its sensitivity to externally applied NO.

## Neurotransmission by NO: general features

Depending on the circuit, NO may be produced pre- or postsynaptically. In many brain areas, the prototypic coupling with postsynaptic NMDA receptors appears to apply. In others, NO may derive from presynaptic axon terminals, much as it does in peripheral nitrergic nerves. In these nerves, the stimulus for NO synthesis is usually the action potential-dependent opening of presynaptic N-type and/or other Ca^2+^ channels, with the resulting NO effecting smooth muscle relaxation (see review by Toda & Herman, 2005). Clues to the shapes of the synaptic NO signals come from experiments where it has been possible to record downstream electrophysiological responses at the single-cell level.

**Fig. 1:**
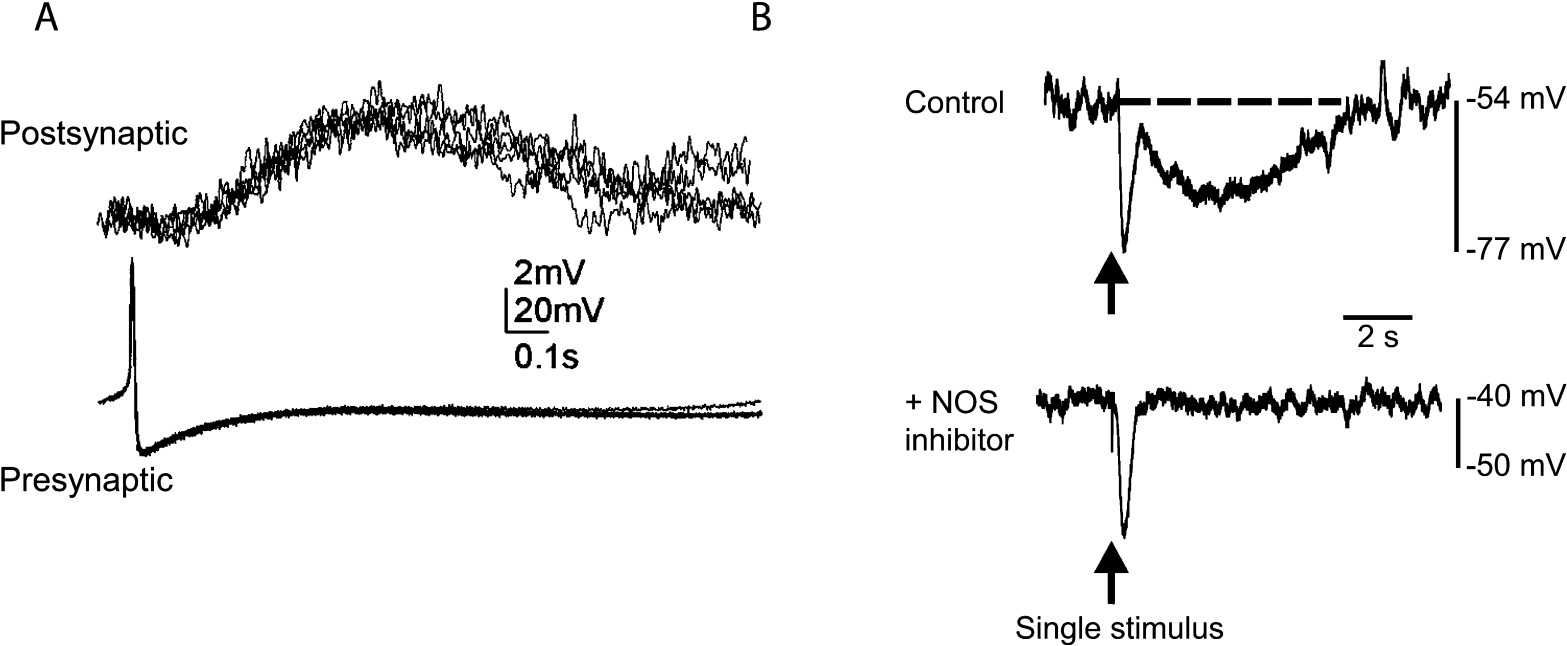
NO-mediated synaptic transmission. (A) In a synaptically coupled pair of *Lymnea stagnalis* neurones, single presynaptic action potentials produce constant-latency, one-to-one unitary EPSPs mediated through NO; five sweeps are superimposed. Modified from Park *et al.* (1998) with permission. (B) Slow inhibitory post-junction potentials in mouse colonic circular muscles induced by afferent nerve stimulation (electrical field stimulation, single pulses, 0.3 ms duration) in the absence and presence of a NOS inhibitor (100 μm l-nitroarginine), showing the slow hyperpolarizing component to be NO-mediated. Note that inhibition of NOS also depolarizes the muscle by ∼14 mV as a result of the removal of a tonic hyperpolarizing NO source. Modified from Hwang *et al.* (2008) with permission.

The first example (Fig. 1A) shows recordings from neurones in the pond snail (*Lymnaea stagnalis*), specifically at synapses between a nitrergic neuron and its partner (Park *et al.*, 1998). Bursts of presynaptic activity resulted in slow NO-mediated excitatory postsynaptic potentials (EPSPs) that could cause postsynaptic spiking whereas single presynaptic action potentials normally had no observable effect. When amplified by increasing the Ca^2+^ and Mg^2+^ concentrations in the bathing medium, however, unitary NO-mediated EPSPs could be visualised. They consisted of slow depolarizations taking off after a fixed delay of ∼200 ms, peaking after ∼0.5 s and decaying back to baseline after ∼1 s. When viewed in this way NO neurotransmission appears quite familiar, being evocative of the slower synaptic transmission occurring through metabotropic GABA or glutamate receptors (Dutar & Nicoll, 1988; Batchelor & Garthwaite, 1997). Moreover, the much more sustained response of the neurones seen after exogenous NO application (Park *et al.*, 1998) suggests that the falling phase of the EPSP is not caused by tachyphylaxis (loss of responsiveness) but probably reflects the earlier dissipation of the NO signal. Hence, the unitary synaptic NO signal is likely to be quite brief.

The second example (Fig. 1B) shows synaptic NO having the opposite effect (hyperpolarization) when released from nitrergic nerves innervating the colon (Hwang *et al.*, 2008). In this case, a single shock to the nerves elicited a biphasic inhibitory potential, an initial rapid phase caused by release of a purine and then a second phase that is the result of NO release. The time-course of the nitrergic potential is a little slower than that of the snail neurone EPSP, peaking after ∼3 s and then falling to baseline after ∼8 s.

**Fig. 2:**
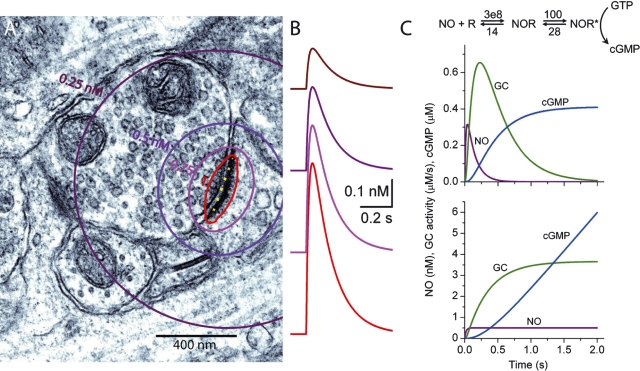
Dynamics of synaptic NO. (A) Superimposed on an electron micrograph of an excitatory synapse (from Kennedy, 2000, with permission) are contours of NO concentrations (inner red ring, 1 nm; outer wine-coloured ring, 0.25 nm) predicted to be formed at steady-state by a 7 × 7 array of active NOS molecules located (approximately) in the postsynaptic density. Each NOS molecule was assumed to generate 20 NO molecules per second, based on the initial rates of NO formation by nNOS reported by Santolini *et al.* (2001) after correcting for temperature. Seven of the source molecules are in the plane of vision at the centre of the contours, the yellow colour signifying 2 nm NO. (B) NO profiles resulting from transient NOS activation at different distances from the sources, colour-coded to the positions of the contours in A. The waveform of the input NOS activity was chosen to resemble the time-course of a unitary NMDA receptor-mediated rise in postsynaptic Ca^2+^, and is superimposable on the output (NO concentration) curves, when scaled. (C) Transduction of an NO transient. The empirical kinetic scheme for NO receptor activation (Garthwaite, 2005) is shown at the top and the resulting activity of receptor-associated guanylyl cyclase activity (GC) and the increase in cGMP are depicted for NO arriving near the middle of the presynaptic terminal (purple contour in A) as a single pulse (upper panel) or during a sustained 2-s period of NOS activity (lower panel). Data are from C.N. Hall and J. Garthwaite (unpublished results).

Although there are no equivalent data dealing with postsynaptically-generated NO, we can begin to envisage what happens. A typical excitatory synapse in the brain would have ∼50 NMDA receptors dispersed over a 400-nm-diameter postsynaptic density (Kennedy, 2000). Taking the extreme situation of each being associated with an nNOS molecule and the nNOS molecules all being maximally active, generating 20 NO molecules each per second, we can see the resulting NO concentration profile that would be set up purely as a result of diffusion of the molecule away from the sources (Fig. 2A). At steady state (with continuous NMDA receptor activation) we expect to find only 1 nm NO just the other side of the synaptic cleft, reducing to 0.25 nm at the periphery of the nerve terminal. A high degree of synapse specificity is implied, although closely neighbouring structures would also be penetrated by low NO concentrations.

When synaptic NMDA receptors are activated by a pulse of glutamate, the resulting local rise in intracellular Ca^2+^ follows a time-course resembling that of the current passing through the membrane, rising to peak in ∼50 ms and then declining to baseline within ∼500 ms (Sabatini *et al.*, 2002). The active Ca^2+^/calmodulin required for nNOS activity has four bound Ca^2+^. On removal of Ca^2+^, unbinding of the first two Ca^2+^ is rapid, causing arrest of NOS activity with a half-time of < 70 ms. Unbinding of the remaining two Ca^2+^ is at least 10-fold slower and dissociation of calmodulin from nNOS slower still, with an estimated half-time of 7 s (Persechini *et al.*, 1996). A scenario for efficacious NO generation, therefore, would be in a recently activated synapse where calmodulin with two Ca^2+^ bound is already associated with nNOS. Synaptic NMDA receptor activation should then result in almost contemporaneous binding of the final two Ca^2+^ and subsequent NO production. The resulting NO pulses predicted at different distances from the source are illustrated in Fig. 2B. There would be no perceptible delay in the peak of the pulse arriving at different parts of the synapse although, of course, the amplitude falls with distance and does not quite achieve that seen with a continuous NO output.

By placing NO receptors at different distances we can gauge how effective they would be at transducing the signals (Fig. 2C, upper panel). Assuming the empirical kinetic scheme drawn up previously (Garthwaite, 2005) and a receptor density the same as is found in platelets (Mo *et al.*, 2004), an NO pulse arriving mid-way through the nerve terminal, peaking at 0.3 nm, would evoke ∼0.4 μm cGMP, an impressive 1000-fold amplification taking place within ∼1 s. Hence, the receptors are beautifully tuned to capture and translate even brief, low-amplitude NO pulses. The simulation ignores hydrolysis of cGMP by phosphodiesterases but, at these concentrations, much of the cGMP would probably bind to signalling proteins in the vicinity which would protect it from degradation (Kotera *et al.*, 2003). The submicromolar affinity of cGMP-dependent protein kinases for cGMP (Wall *et al.*, 2003) provides a mechanism for such an NO pulse being biologically significant and it is notable that the time-courses of the simulated responses fall reasonably well in line with those of the potential changes recorded electrophysiologically (Fig. 1), bearing in mind the steps downstream from cGMP that will help shape the physiological response.

It is probably unrealistic to imagine all synaptic NMDA receptors being momentarily active together but the simulation in Fig. 2C (lower panel) shows that if there were repeated stimuli leading to a 2-s period of continuous NOS activity, cGMP may accumulate into the low micromolar range (a 10 000-fold amplification over the NO concentration), meaning that many fewer NMDA receptors would need to be active to generate a biological response. The temporal summation of the cGMP concentration in this scenario helps explain why NO-mediated transmission is generally seen to operate most effectively following short periods of higher frequency activity.

That endogenous NO should be acting at synapses in concentrations of only ∼1 nm, which is a tenth of the concentration needed to activate cellular receptors by 50% (see above), may seem strange. However, we do not have to rely merely on these theoretical calculations for the evidence. The value is consistent with vascular relaxation being maximal at NO concentrations near the base of the concentration–response curve for cGMP generation (Mullershausen *et al.*, 2006), with cGMP-dependent phosphorylation being triggered by subnanomolar NO concentrations (Mo *et al.*, 2004), and with data from NO receptor-knockout mice showing that NO can still relax blood vessels when 94% of the associated guanylyl cyclase activity has been eliminated (Mergia *et al.*, 2006). All these experimental findings suggest that cells have a large receptor reserve, ensuring that low-amplitude NO signals are captured and transduced.

As well as mediating point-to-point transmission at synapses, there may be situations in which neuronally derived NO performs as a ‘volume’ transmitter. There are two anatomical scenarios where volume transmission is most likely. One is where there is a plexus of NO-releasing nerve fibres that have a density and degree of synchronous activity that will allow a regional ‘cloud’ of NO to be formed (Philippides *et al.*, 2005). The other is when there is an anatomically appropriate segregation of NO sources and targets, as exemplified in the insect brain by the so-called mushroom body, which is involved in associative learning. In the ‘stalk’ of the mushroom body, the NO sources were located in a sheath of nerve fibres (∼30 μm thick) which enwrapped a ∼30-μm-diameter core of NOS-negative axons expressing the NO receptors; the design features, together with a theoretical analysis, suggested that the outer sleeve could act like a heating jacket, radiating NO to the inner core to generate a relatively uniform concentration within it (Ott *et al.*, 2007). Volume transmission by NO has attractions for certain theories of learning in which alterations in synaptic strength are governed by the combination of the ambient NO concentration in the region and the coincident synaptic activity (Montague & Sejnowski, 1994). An alternative to neurones as the source of ambient NO in this setting might be the capillary endothelial cells (see below).

## Acute synaptic actions of NO

A special property of NO compared with conventional neurotransmitters is its free diffusion through aqueous and lipid environments, so it is not possible to predict where it will act after being synthesised in either pre- or postsynaptic sites from the standpoint of diffusion alone (Fig. 2A). Indeed, a special advantage of a messenger such as NO would be that it provides a simultaneous signal to both pre- and postsynaptic elements, of probable importance in coordinating responses on the two sides of the synapse. A common assumption in the literature is that if an NO-mediated response is inhibited by a scavenger that remains extracellular, such as haemoglobin, NO must be acting intercellularly. This assumption is quite wrong. NO travels randomly and surprisingly quickly. With a tissue diffusion coefficient of 848 μm^2^/s (Liu *et al.*, 2008) the average NO molecule travels ∼0.8 μm (twice the diameter of the postsynaptic density) in 100 μs (Lancaster, 1997) which means that, on biological time-scales, it will constantly diffuse in and out of the NO-generating compartment. Consequently, an efficient extracellular scavenger will inevitably deplete both intracellular and extracellular NO.

Generally speaking, however, NO does appear to signal to the opposite synaptic partner, as in the examples illustrated (Fig. 1), but both pre- and postsynaptic actions are feasible (irrespective of the site of generation), depending on location of the receptors. The actions of NO on either structure, despite employing the same transduction mechanism (cGMP), obey no general rules and, even when a similar effect is observed, the underlying mechanisms may be different. In one of the sample synapses (Fig. 1B), the hyperpolarizing postsynaptic potential observed on stimulation of nitrergic nerves was ascribed to the activation of background K^+^ channels, a major contributor being a member of the two-pore-domain K^+^ channel family, TREK-1, with the effect of NO being transduced through serine phosphorylation by PKG (reviewed in Sanders & Koh, 2006). TREK-1 channels are widely expressed in the brain, often in association with GABAergic neurones (Fink *et al.*, 1996; Hervieu *et al.*, 2001) and it will be interesting to know whether NO–cGMP operates through these channels elsewhere. The EPSP in the snail neurones (Fig. 1A) appears to be caused by closure of K^+^ channels, although this was not explicitly examined (Park *et al.*, 1998). A similar NO-mediated EPSP has been recorded in an *Aplysia* neurone modulating the feeding circuit where it was shown that, through cGMP, synaptically released NO inhibited background K^+^ channels (Jacklet & Tieman, 2004).

Together, the two examples encapsulate observations made at many different CNS synapses. A large literature describes various reversible effects of NO and/or cGMP on neuronal excitability and/or synaptic transmission, the effects being generally classifiable as excitatory or inhibitory. It is useful to give a few examples, selected on the basis of evidence that endogenous NO engages the same mechanism. This is a particularly important criterion here, bearing in mind that exogenous NO (or its method of delivery) may produce unphysiological effects and that too much cGMP may also be unphysiological, leading, for example, to inadvertent activation of cAMP-dependent protein kinases (Sausbier *et al.*, 2000). In some cases, endogenous activity is promoted by administration of the NOS substrate l-arginine, and it is relevant that the l-arginine concentration in the cerebrospinal fluid is ∼20 μm (Martens-Lobenhoffer *et al.*, 2007) but is normally omitted from solutions used to incubate *in vitro* preparations, possibly resulting in a suppression of NO-mediated transmission in some experiments. It should be cautioned, however, that l-arginine may have effects unrelated to NOS activity (Hentall, 1995; Rivadulla *et al.*, 1997). Frequently missing are measurements of NO itself but, unfortunately, there are no reliable methods yet for directly measuring endogenously generated NO in tissues with the necessary sensitivity or spatial and temporal precision (reviewed in Keynes & Garthwaite, 2004; Wang *et al.*, 2006a). Nevertheless, useful data at a more gross level have been obtained from certain electrode designs (e.g. Shibuki & Kimura, 1997) but many of those that have been used suffer from suspect specificity and inadequate sensitivity.

### Postsynaptic actions

Earlier *in vivo* experiments on the lateral geniculate and ventrobasal nuclei of the thalamus showed that NO had a marked facilitatory effect on neuronal responses to natural stimuli (light and whisker stimulation, respectively), as well as on the excitatory effect of locally administered glutamatergic agonists (Do *et al.*, 1994; Cudeiro *et al.*, 1996; Shaw *et al.*, 1999). A major source of the NO in these nuclei is in afferent cholinergic fibres, suggesting that presynaptically derived NO is at work whereas the downstream enhancement of excitation was probably postsynaptic, putatively through direct engagement by cGMP of HCN channels that are prominent in the thalamus (Shaw *et al.*, 1999). A similar general enhancing effect of NO was seen in the visual cortex, where inhibition of NO synthesis depressed responses to exogenous agonists and to visual stimuli (Cudeiro *et al.*, 1997; Kara & Friedlander, 1999) and in the somatosensory cortex, where NO participated in the ‘wake-up’ electrical activity observed following stimulation of the basal forebrain cholinergic afferents (Marino & Cudeiro, 2003). In the striatum *in vivo* too, NO (probably derived from local interneurones that are rich in nNOS) modulated excitation of medium spiny neurones in such a way as to enhance EPSPs (West & Grace, 2004). Its effect here was associated with membrane depolarization, perhaps brought about by cGMP suppressing K^+^ conductances.

Beyond these pioneering *in vivo* studies, NO–cGMP has also been found to have depolarizing actions on several different types of central neurone *in vitro*, including a population of paraventricular neurones (Bains & Ferguson, 1997), striatal cholinergic neurones (Centonze *et al.*, 2001), trigeminal motoneurones (Abudara *et al.*, 2002) and optic nerve axons (Garthwaite *et al.*, 2006). HCN channels were implicated as the transducers in the latter two instances. In a pituitary cell line, endogenous NO elicited a cGMP-dependent inhibition of K^+^ channels, an effect of which would also be membrane depolarization, although cytosolic Ca^2+^ oscillations were actually measured in the experiments (Secondo *et al.*, 2006). Alternatively, CNG channel activation following NO-evoked cGMP accumulation may produce excitatory postsynaptic responses in central neurones, as was shown first in a population of retinal ganglion cells (Ahmad *et al.*, 1994; Kawa & Sterling, 2002) and, more recently, in medial vestibular nucleus neurones (Podda *et al.*, 2008), but the engagement of this pathway by endogenous NO has not yet been demonstrated.

Postsynaptic inhibitory effects of NO–cGMP on neuronal firing, or hyperpolarisations, have also been reported (Schmid & Pehl, 1996; Rauch *et al.*, 1997; Xu *et al.*, 1998; Riediger *et al.*, 2006; Sardo *et al.*, 2006). Potential mechanisms include the activation of various classes of K^+^ channel (Han *et al.*, 2006; Kang *et al.*, 2007; Mironov & Langohr, 2007; Chai & Lin, 2008) but, again, there is little evidence yet for endogenous NO acting in this way (but see Cudeiro *et al.*, 1997, for an *in vivo* example).

### Presynaptic actions

An altogether different response to NO is exemplified by results from the paraventricular nucleus, which is important in autonomic and endocrine homeostasis. NMDA or angiotensin was found to cause a barrage of GABAergic inhibitory postsynaptic potentials (IPSPs) in the magnocellular neurones of this region that was mediated, at least in part, by endogenous NO exciting GABAergic neurones (Bains & Ferguson, 1997; Latchford & Ferguson, 2003). These results were extended to spinally projecting neurones within this nucleus, whose firing was regulated by NO modulating the GABAergic tone (Li *et al.*, 2002, 2003). In such neurones, studies of miniature GABAergic IPSPs (recorded in the presence of tetrodotoxin to prevent circuit-based activity) strongly suggested that NO was acting on presynaptic terminals to increase the probability of vesicular GABA release (Li *et al.*, 2002), a mechanism that has been attributed to NO acting through cGMP and PKG (Li *et al.*, 2004). Presynaptic voltage-gated K^+^ channels, specifically subtypes Kv1.1 and 1.2 but perhaps others as well, have recently been implicated (Yang *et al.*, 2007). Where studied in the accessible calyx of Held nerve terminals in the brainstem, Kv1 channels appear to be located in the transition zone just before the terminal itself and to regulate terminal excitability in such a way as to suppress aberrant action potential firing and associated neurotransmitter release (Dodson *et al.*, 2003; Ishikawa *et al.*, 2003). Possibly, therefore, cGMP-dependent phosphorylation directly or indirectly inhibits the channels, resulting in enhanced GABA release. Interestingly, Kv3 channels, which affect transmitter release by shaping the presynaptic action potential, have been found, when expressed in CHO cells, to be suppressed by the NO–cGMP pathway, not through direct phosphorylation by PKG but through the intermediary of a phosphatase, which probably removes a phosphate group from the channel protein that normally promotes activity (Moreno *et al.*, 2001). Should this mechanism occur in nerve terminals, spike broadening, leading to increased transmitter release, would be anticipated (Ishikawa *et al.*, 2003).

While these investigations focus on K^+^ channel inhibition as a mechanism of increasing presynaptic transmitter release, K^+^ channel activation may paradoxically improve transmitter release as well. Specifically, stimulation of Ca^2+^-activated K^+^ channels by cGMP-dependent phosphorylation enhanced presynaptic spiking by increasing the spike afterhyperpolarization, allowing more Na^+^ channels to recover from inactivation which, by facilitating spike conduction, increased Ca^2+^influx in response to trains of stimuli (Klyachko *et al.*, 2001).

One shortcoming in many studies of synaptic NO signalling is ignorance of exactly where the NO comes from and where it acts. Considerations based on individual synapses (Fig. 2) would predict that the sources and targets would need to be close together for the signalling pathway to work, although summation of signals extrasynaptically would be plausible if groups of synapses were simultaneously active (Philippides *et al.*, 2005; Hall & Garthwaite, 2006). According with this idea, high resolution studies of hippocampal CA1 pyramidal neurones showed that, in a population of synapses, NO receptors were located in the active zone in excitatory nerve terminals whereas nNOS was concentrated in juxtaposed dendritic spines just below the plasma membrane, an arrangement well suited to a retrograde messenger role for NO at these synapses (Burette *et al.*, 2002). Unexpectedly, in the same neurones an additional line of communication has materialised from nNOS also being found in the postsynaptic densities of GABAergic terminals (on somata, dendrites and axon initial segments) with the NO receptors being presynaptic (Szabadits *et al.*, 2007). The NO receptor at GABAergic synapses was, interestingly, the α1β1 isoform whereas the α2β1 receptor, which associates with pre- and/or postsynaptic scaffold proteins (see above), appeared to service the excitatory synapses. This arrangement immediately suggests a new type of retrograde NO signalling wherein local rises in cytosolic Ca^2+^ as a result of back-propagating action potentials or of local synaptic input activates postsynaptic nNOS and the NO then signals to abutting inhibitory nerve terminals to influence GABA release (Szabadits *et al.*, 2007). Supporting this line of communication being functional, when studied in the presence of a cholinergic agonist, depolarization (for 1 s) of the postsynaptic neurone caused a transient (∼20 s) suppression of GABAergic inhibitory postsynaptic currents (IPSCs), an effect mediated through NO receptors and cGMP (Makara *et al.*, 2007), with endocannabinoids also being intimately involved (see below).

In these experiments, spontaneous quantal GABAergic IPSCs (recorded in the presence of tetrodotoxin) were unaffected by NOS or NO receptor blockade (Makara *et al.*, 2007), indicating that cGMP suppressed action potential-dependent GABA release ultimately, perhaps, by inhibiting presynaptic voltage-gated Ca^2+^ channels. In this respect there is a large literature dealing with NO–cGMP and Ca^2+^ homeostasis in cells of relevance to both its pre- and postsynaptic actions (reviewed in Garthwaite & Boulton, 1995; Clementi, 1998; Ahern *et al.*, 2002; Grassi *et al.*, 2004) but no general rules: in some cells Ca^2+^ currents are inhibited, in others they are enhanced; sometimes Ca^2+^ release from internal stores is reduced, elsewhere it is increased. Regarding the latter, recordings in the nucleus of the solitary tract found that, via cGMP, low concentrations of exogenous NO reversibly potentiated both glutamatergic EPSPs and GABAergic IPSPs, apparently through a presynaptic mechanism (Wang *et al.*, 2007). In the case of the IPSPs, NO–cGMP seemed to act by evoking Ca^2+^ release from presynaptic ryanodine-sensitive stores through the intervention of cyclic ADP ribose (Wang *et al.*, 2006b), whose generation can be stimulated by PKG phosphorylating the synthesising enzyme (Willmott *et al.*, 1996). Elsewhere, NO may enhance spontaneous neurotransmitter release by the action of cGMP on presynaptic CNG channels, leading to a raised intraterminal Ca^2+^ concentration (Savchenko *et al.*, 1997; Murphy & Isaacson, 2003). A major NO–PKG transduction pathway in smooth muscle and platelets is through the IP3 receptor-associated protein IRAG which, when phosphorylated by PKG1β, inhibits Ca^2+^ release from IP3-sensitive stores (reviewed in Hofmann *et al.*, 2006). However, IRAG appears to have a minor presence in the brain, the mRNA being prominent only in thalamic relay nuclei (Geiselhoringer *et al.*, 2004) where, perhaps significantly, PKGII is also concentrated (El-Husseini *et al.*, 1999; de Vente *et al.*, 2001).

### Interaction with other signalling pathways

Apart from these serial signalling cascades influencing primary synaptic neurotransmission, the interplay between NO–cGMP and other neuromodulators introduces another layer of regulation. This is presaged by findings in the peripheral nervous system at junctions between nitrergic nerves and effector organs, where a multitude of interactions with cholinergic, adrenergic, purinergic and peptidergic nerves, often presynaptic, has been described (reviewed in Toda & Okamura, 2003; Toda & Herman, 2005). Similarly, based on *in vivo* sampling of the extracellular fluid, myriad interactions with other transmitters in the brain are to be expected (reviewed in Prast & Philippu, 2001), although details of what may be happening at the level of the synapse are sparse. In the pond snail, serotonin transmission was markedly (up to 80%) dependent on endogenous NO acting postsynaptically through cGMP and PKG, perhaps bringing about phosphorylation of the serotonin receptor (Straub *et al.*, 2007). There is also long-standing evidence for an interaction between NO, cGMP and acetylcholine in the brain that remains poorly understood (reviewed by de Vente, 2004). One intriguing link with acetylcholine may involve the endocannabinoids. These are fatty acid derivatives that act in synapses somewhat like NO, in that they are generated enzymatically in response to a rise in Ca^2+^, usually postsynaptically, and then act retrogradely on presynaptic CB1 receptors, typically to depress neurotransmitter release (reviewed in Hashimotodani *et al.*, 2007). In the vertebrate (lizard) neuromuscular junction, endocannabinoid release underlay the transient suppression of acetylcholine release produced by activation of muscarinic (M3) receptors but, in order for the pathway to be effective, NO–cGMP and associated PKG were needed (Newman *et al.*, 2007). NO on its own did not affect acetylcholine release (Graves *et al.*, 2004), so there seems to be a step occurring in the presynaptic nerve terminal where endocannabinoid and NO signalling cascades meet. Somewhat similarly, the depression of GABAergic IPSCs brought about by depolarizing hippocampal neurones in the presence of a cholinergic agonist depended on both NO and endocannabinoids, the two of them apparently being produced postsynaptically and acting presynaptically (Makara *et al.*, 2007). Unlike in the lizard neuromuscular junction, cannabinoids could depress transmission on their own but, in the presence of the cholinergic agonist, the NO–cGMP and cannabinoid pathways converged, apparently at the presynaptic location (where CB1 receptors and NO-evoked cGMP accumulation were located) to elicit the transient depression of GABAergic transmission. Further interplay between endocannabinoids and NO can be found in relation to long-lasting alterations in synaptic efficacy (see below and Sergeeva *et al.*, 2007; Kyriakatos & El Manira, 2007).

## NO and neuroplasticity

There has been much interest in an involvement of NO in long-term changes in synaptic strength, initially because its diffusible nature and link with NMDA receptors made it an appealing candidate for a retrograde trans-synaptic messenger, relaying information about NMDA receptor activity to the presynaptic terminal to coordinate alterations in transmitter release (Garthwaite *et al.*, 1988). Despite some confusing results early on, it is now established that the NO–cGMP pathway plays a role in long-term potentiation (LTP) or long-term depression (LTD) at many synapses throughout the CNS, and even at the neuromuscular junction. As the topic has been reviewed many times before, both generally (Garthwaite & Boulton, 1995; Holscher, 1997; Prast & Philippu, 2001; Susswein *et al.*, 2004) and with respect to specific brain regions (Daniel *et al.*, 1998; Hawkins *et al.*, 1998; Centonze *et al.*, 1999; Grassi & Pettorossi, 2001; Hartell, 2002), the emphasis here will be on mechanisms and, as with the acute synaptic actions of NO (above), these can be pre- and/or postsynaptically located, or they can involve more general alterations in neuronal excitability.

### Postsynaptic plasticity

One of the most developed instances of NO participating in plasticity is with LTD in the cerebellum, which has long been considered part of motor learning behaviour and which occurs when a powerful excitatory input to Purkinje cells from the climbing fibres is repeatedly active just after another, from parallel fibres. The climbing fibre input is viewed as an error signal which dampens input from parallel fibres that are inappropriately active just beforehand (reviewed by Ito, 2001). According to the current model, NO is produced either in parallel fibres themselves (Shibuki & Kimura, 1997) or via NMDA receptor activity in interneurones simultaneously receiving parallel fibre excitation (Shin & Linden, 2005) and acts postsynaptically at parallel fibre synapses to raise cGMP and thence activate PKG. Purkinje cells are enriched in the PKG substrate, known as G-substrate, that, on phosphorylation, functions as a phosphatase inhibitor (Endo *et al.*, 1999). This, together with ongoing protein kinase C activity, leads to persistent phosphorylation of AMPA receptors at a particular serine residue, which disrupts AMPA receptor clusters and favours receptor endocytosis (Launey *et al.*, 2004; Steinberg *et al.*, 2006). Consistent with the mechanism being behaviourally significant, knocking down PKG1α specifically in Purkinje cells impaired LTD and introduced a deficit in a motor learning behaviour (adaptation of the vestibulo-ocular reflex) although their general motor performance was normal (Feil *et al.*, 2003). Endocannabinoids figure in cerebellar LTD as well and it appears that NO functions downstream of this pathway because endocannabinoid-induced LTD could be blocked by NOS inhibition, suggesting that endocannabinoids may somehow promote the NO synthesis needed for LTD (Safo & Regehr, 2005). A long-term potentiation of NO release from the parallel fibres following tetanic stimulation has been described (Kimura *et al.*, 1998) but this depended on cAMP, whose levels are usually reduced by endocannabinoids (reviewed in Hashimotodani *et al.*, 2007). Even so, there are precedents for endocannabinoid CB1 receptors being coupled to increased NO synthesis (Poblete *et al.*, 2005; Romano & Lograno, 2006).

Long-term potentiation in the hippocampus also involves changes in postsynaptic AMPA receptor density, but in the opposite direction (reviewed in Collingridge *et al.*, 2004) and NO–cGMP may play a part in this process. A key AMPA receptor subunit required for NMDA receptor-dependent LTP is the GluR1 subunit, whose insertion into the synapse depends on complex interactions with synaptic scaffold proteins and protein phosphorylation. In dissociated hippocampal cultures brief application of glutamate elicited an enduring potentiation of spontaneous glutamatergic excitatory postsynaptic currents (EPSCs) comprising a rapid enhancement in frequency, originating presynaptically, and an increase in amplitude, associated with increased numbers of postsynaptic protein clusters containing GluR1 (Antonova *et al.*, 2001). A surprising finding was that the increase in GluR1 clusters required PKG and could be replicated by exposure to 8-Br-cGMP (Wang *et al.*, 2005). In a comprehensive series of experiments, Serulle *et al.* (2007) have now discovered a key participant to be PKGII. Binding of cGMP led to the formation of a complex between PKGII and GluR1 and the phosphorylation of GluR1 on a serine residue (serine-845) that facilitates its delivery to extrasynaptic sites, priming insertion into the synapse. Previously, serine-845 phosphorylation had been linked with cAMP but Serulle *et al.* (2007) showed in cultured hippocampal neurones that it also happens in response to exogenous NO, and to NMDA receptor activity in a NOS- and PKG-dependent manner. Increased cell surface GluR1 expression correlated with changes in synaptic transmission that shared the same NO–cGMP–PKG-dependent properties.

The receptor clustering seen in these experiments is reminiscent of that occurring in the developing neuromuscular junction, where agrin secreted from active motor nerve terminals induces, through the NO–cGMP–PKG pathway, the formation of postsynaptic acetylcholine receptor clusters (reviewed in Godfrey & Schwarte, 2003). However, Serulle *et al.* (2007) were able show that blocking PKGII selectively using a dominant negative fragment also reduced LTP in adult mouse hippocampal slices, indicating that the mechanism may not be confined to the relatively immature circuitry of the tissue culture model.

These two examples of NO–cGMP effecting opposite changes in postsynaptic AMPA receptor density at two different synapses may help explain the participation of NO in LTP and LTD elsewhere. However, other mechanisms must also at be work. For hippocampal LTP to persist beyond the first hour or so, RNA and protein synthesis are needed. In slice preparations, it has been shown that NO–cGMP–PKG resulted in the phosphorylation of the transcription factor CREB (cAMP response element binding protein) in the cell bodies of postsynaptic neurones by a mechanism involving Ca^2+^ release from ryanodine-sensitive stores (Lu *et al.*, 1999; Lu & Hawkins, 2002), implicating cyclic ADP ribose as an intermediary. CREB regulates the expression of many different genes. There are also several other ways by which cGMP can directly and indirectly regulate gene expression in cells. In all, > 60 RNA species have so far been shown to be increased or decreased by cGMP in various cell types (reviewed in Pilz & Broderick, 2005).

### Presynaptic plasticity

The first direct evidence for NO persistently augmenting neurotransmitter release came from recordings showing that exogenous NO, added at 5–10 nm, elicited an enduring increase in the frequency of spontaneous miniature EPSPs in cultured hippocampal neurones (O’Dell *et al.*, 1991). Subsequent work extended this observation to provide a convincing case for NO serving as a retrograde messenger at these synapses in response to brief tetanic stimulation of the presynaptic neurone, and that it acts through cGMP and PKG (Arancio *et al.*, 1995, 2001), leading to the rapid (within a minute) formation of new clusters of presynaptic proteins, coordinating with the slightly later appearance of new postsynaptic GluR1 clusters (Wang *et al.*, 2005). A protein well known in the cardiovascular field as a PKG substrate, vasodilator-stimulated phosphoprotein (VASP), together with RhoA, a member of the Rho GTPase family, apparently contributed to the clustering on both sides of the synapse, suggesting a cytoskeletal involvement, with the conversion of the clusters into functional units, perhaps under the control of CaMKII activity (Ninan & Arancio, 2004; Wang *et al.*, 2005). The results are consistent with the functioning of VASP in filopodial dynamics (Dwivedy *et al.*, 2007) and with evidence that NO mediates NMDA receptor-dependent growth of presynaptic protrusions and the remodelling of presynaptic varicosities in hippocampal slice cultures (Nikonenko *et al.*, 2003).

Good evidence from other synapses also implicates a presynaptic site of action of NO although, in most cases, the mechanisms remain to be explored (Wu *et al.*, 1997; Grassi & Pettorossi, 2000; Volgushev *et al.*, 2000; Hardingham & Fox, 2006; Sjostrom *et al.*, 2007). In rat rostral ventral medulla neurones, endogenous NO was found to operate though cGMP, PKG and presynaptic N-type Ca^2+^ channels to potentiate glutamate release for 10–20 min (Huang *et al.*, 2003). In the cerebellar parallel fibre–Purkinje cell synapse, NO was required for a presynaptic form of LTP which, curiously, depended on cAMP but not cGMP (Jacoby *et al.*, 2001) and which reflected an augmentation of action potential-evoked presynaptic Ca^2+^currents (Qiu & Knopfel, 2007). LTP at the cerebellar mossy fibre–granule cell synapse, however, engaged the NO–cGMP pathway to increase the presynaptic terminal excitability (Maffei *et al.*, 2003). NO–cGMP-dependent potentiation of inhibitory, presumed glycinergic (Wu & Dun, 1996) and GABAergic (Nugent *et al.*, 2007), synapses through the presynaptic route have also been reported. Finally, NO may also contribute to LTD by suppressing presynaptic excitatory transmitter release cGMP-dependently, as has been observed in the hippocampus (Zhang *et al.*, 2006b) and the neuromuscular junction (Wang *et al.*, 1995; Etherington & Everett, 2004).

A role for NO in plastic changes of other neurotransmitter systems is also likely. For example, it has been found that PKG-dependent phosphorylation of a threonine residue on the serotonin transporter enhanced serotonin uptake (Ramamoorthy *et al.*, 2007), an effect that may contribute to the lowering of extracellular serotonin brought about by the NO–cGMP pathway observed in the hypothalamus (Kaehler *et al.*, 1999), and to obsessive–compulsive disorder in humans (Zhang *et al.*, 2007a). Alternatively, nNOS may physically interact with the serotonin transporter in such a way as to reduce its cell surface expression, which should increase extracellular serotonin levels (Chanrion *et al.*, 2007).

### Plasticity of intrinsic excitability

Plasticity can also be expressed outside the synapse, through alterations in intrinsic neuronal excitability (reviewed in Daoudal & Debanne, 2003) and here too NO may be involved. In the cerebellum, Purkinje cell firing became moderately higher, but in a sustained manner, in response to NO–cGMP (Smith & Otis, 2003). A more pronounced effect was observed in *Aplysia* sensory neurones which, much like in mammals, become hyperexcitable (decreased threshold for action potential generation, and increased depolarization-induced and spontaneous firing) following injury to their axons (Sung *et al.*, 2004). Underlying the hyperexcitability was an upregulation of nNOS, stimulation of which, apparently at the level of the axon, led to local activation of PKG which then was transported to the neuronal cell body. Here it phosphorylated mitogen-activated protein kinase which then entered the nucleus to initiate transcriptional activity. As well as providing a mechanism for enduring hyperexcitability, the results give an example of how NO acting remotely on neuronal processes can engage the gene expression machinery in the cell body. Extending the results to mammals, it was found that compression injury to rat dorsal root ganglion neurones, or simply dissociating the neurones, resulted in them adopting a hyperexcitable state that was maintained by cAMP and cGMP acting through their respective kinases (Song *et al.*, 2006; Zheng *et al.*, 2007). Furthermore, in hypoglossal motoneurones, while brief NO exposure normally generated only a small cGMP-dependent depolarization, upregulation of nNOS as a result of nerve injury, or prolonged (4 h) exposure to exogenous NO, led to sustained hyperexcitability through PKG-mediated inhibition of resting K^+^ currents, particularly those produced by the pH-sensitive, two-pore-domain TASK-like channels (Gonzalez-Forero *et al.*, 2007), channels that are widely distributed in the CNS (reviewed by Bayliss *et al.*, 2003). The gradual onset of the NO-mediated suppression of the channels suggests a mechanism operating through trafficking or gene/protein expression (Gonzalez-Forero *et al.*, 2007).

Although these last two examples are of specific relevance to the development of heightened pain sensitivity and other responses to injury, they may also exemplify changes enacted under normal conditions to contribute, along with synaptic changes, to the development of activity-dependent alterations in excitability that link cellular plasticity to learning and memory formation (Daoudal & Debanne, 2003).

### Roles of individual NO receptors

The development of mice lacking specific NO receptor subunits (Friebe *et al.*, 2007) offers new opportunities for understanding the roles of each receptor isoform in neuroplasticity and other phenomena. Fascinatingly, it has so far been shown that knocking out either the α1β1 or α2β1 virtually abolished NMDA receptor-dependent LTP in the visual cortex, with rescue being effected by an exogenous cGMP derivative in both cases (Haghikia *et al.*, 2007). Why both isoforms are essential remains a mystery. Perhaps one (α2β1) transduces the NO signal associated with NMDA receptor activity at synapses and the other (α1β1) transduces the signal from endothelial cells (see below). The precise distribution of the isoforms in the cortex remains to be determined but, in the hippocampus, they were found in different cells, α1β1protein appearing to be exclusive to a population of GABAergic interneurones, whereas the mRNA for α2β1 was confined to pyramidal neurones (Szabadits *et al.*, 2007).

## Other lines of communication involving NO

### Glia as NO targets

The highest concentration of cGMP in the cerebellum is in astrocytes (de Vente *et al.*, 1990; Southam *et al.*, 1992; Southam & Garthwaite, 1993), because these cells have an extremely low phosphodiesterase activity, some 6000- to 9000-fold less than in platelets (Garthwaite, 2005), which allows cGMP to accumulate to near millimolar concentrations on persistent activation of NO receptors (Bellamy & Garthwaite, 2001a). Immunocytochemical studies have also detected astrocytic cGMP staining in response to NO in other brain regions (de Vente *et al.*, 1998). Astrocyte processes frequently enwrap synapses and so could be within range of synaptically generated NO. The correlation of cerebellar cGMP levels *in vivo* (largely reflecting glial cGMP) with motor activity (Wood, 1991) also suggests a link with synaptic function. The slow kinetics of cGMP degradation in cerebellar astrocytes could mean that cGMP provides a time-averaged readout of ongoing synaptic activity. Alternatively, it could allow cGMP to diffuse intracellularly to targets distant from the site of synthesis. One report found that, in the cerebellum, parallel fibre stimulation led to a NO-mediated increase in Ca^2+^ in the Bergmann glia (Matyash *et al.*, 2001) but this was not replicated in other laboratories (Beierlein & Regehr, 2006; Piet & Jahr, 2007). Nevertheless, in forebrain cultures, brief (100 ms) puffs of NO elicited a rise in glial Ca^2+^ and the propagation of intercellular Ca^2+^ waves, due to cGMP–PKG promoting release from ryanodine-sensitive stores (Willmott *et al.*, 2000). In cultured astrocyte-like cells (tanycytes) from the median eminence of the hypothalamus, where neurosecretory terminals are normally found close to blood vessels, co-culture with endothelial cells from the same region caused remodelling of the actin cytoskeleton through NO–cGMP and evidence was obtained in the intact tissue that endogenous NO helped position the nerve terminals next to the capillaries to facilitate delivery of secreted hormone, and hence regulate reproductive function (De Seranno *et al.*, 2004). In other astrocytes also, NO–cGMP (through PKG) regulated the expression of glial fibrillary acidic protein, the principal intermediate filament of astrocytes (Brahmachari *et al.*, 2006), and altered cytoskeletal dynamics (Boran & Garcia, 2007).

### Neurovascular communication

Cerebral blood flow is closely linked with neuronal activity. Larger blood vessels are supplied with nitrergic nerves derived mainly from the pterygopalatine ganglion, activity in which results in NO release, vasodilatation and increased blood flow (reviewed by Toda & Okamura, 2003). The first evidence linking local synaptic activity to NO-mediated vascular relaxation came with the demonstration that the NMDA-induced dilatation of cerebral pial arterioles *in vivo* can be blocked by NOS inhibitors (Faraci & Breese, 1993; Faraci & Brian, 1995). NMDA application in hippocampal slices also resulted in NO-dependent vasodilatation of microvessels, adding support to the existence of a local mechanism (Lovick *et al.*, 1999). However, this response to NMDA, and that occurring *in vivo* (Faraci & Breese, 1993), was nullified by tetrodotoxin, implying that the NO causing the vasodilatation is not produced directly by NMDA receptor stimulation but through a secondary, action potential-dependent mechanism. In the cerebellum and cortex, this may involve firing in specific classes of nNOS-containing GABAergic interneurone that innervate the microvasculature (Yang *et al.*, 2000; Cauli *et al.*, 2004; Rancillac *et al.*, 2006), with NO working alongside several other local constrictor and dilator molecules, including peptides and prostanoids (reviewed by Iadecola, 2004).

### Vasculoneuronal communication

**Fig. 3:**
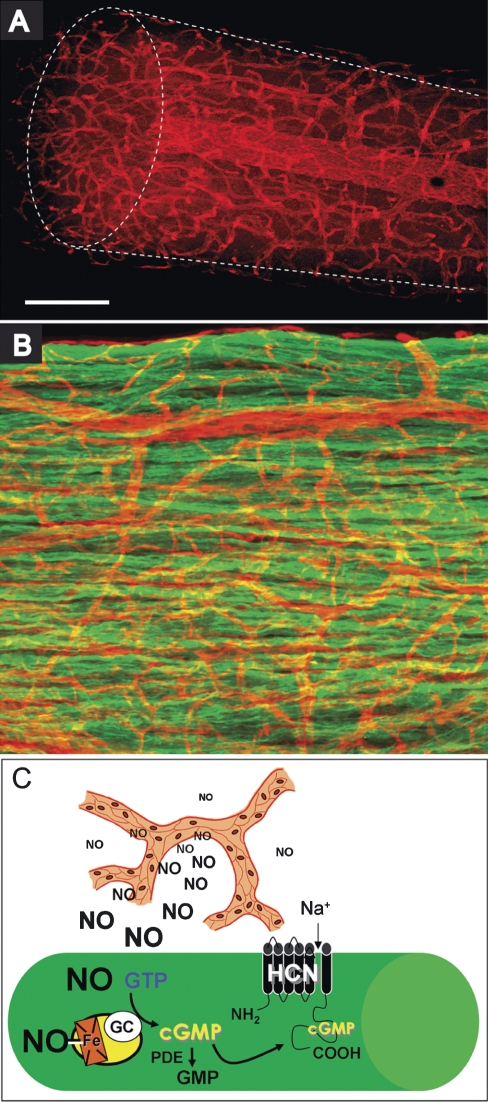
Signalling from capillaries to axons in optic nerve. (A) Whole-mount preparation of 10-day old rat optic nerve immunostained for eNOS. The dotted line depicts the shape of the nerve, including its cut end (ellipse). The image is taken from Garthwaite *et al.* (2006) with permission. (B) Confocal micrograph of optic nerve co-stained for neurofilament-68 (green, identifying axons) and eNOS (red) in the optic nerve (also whole-mount preparation). The picture was kindly provided by Dr G. Garthwaite. (C) Cartoon of the proposed mechanism whereby NO generation from eNOS in the capillary circulation persistently depolarizes axons by raising the levels of cGMP which then acts on HCN channels (from Bartus *et al.*, 2007 with permission). Scale bar (in A), 200 μm (A), 100 μm (B).

The role of endothelial eNOS in relaxing vascular smooth muscle is firmly established, but most of the eNOS in the brain lies in the capillary circulation which, by definition, is devoid of smooth muscle layers (although some modified smooth muscle cells, or pericytes, are present) raising the possibility that capillary eNOS serves another function. Blood vessels are usually far from the mind of the synaptically orientated neuroscientist, yet any region of the brain parenchyma is, at most, only a typical cell diameter (25 μm) away from a capillary endothelial cell (Pawlik *et al.*, 1981). Furthermore, the three-dimensional geometry of the capillary circulation would be just as well suited for delivering NO globally to the tissue as it is for delivering O_2_ (Fig. 3A and B).

The first evidence for endothelial NO influencing parenchymal cells was from the liver where it potentiated Ca^2+^ signalling in hepatocytes (Patel *et al.*, 1999). In the CNS, electrophysiological recordings from the isolated optic nerve showed that there was an endogenous source of NO that persistently depolarized the axons by activating HCN channels; the source was tracked down to being eNOS in the capillary endothelial cells (Garthwaite *et al.*, 2006) (Fig. 3C). In hippocampal slices also, eNOS was the isoform responsible for the tonic, low level of NO (estimated to be ∼0.1 nm) that, along with the burst of NO associated with synaptic NMDA receptor activation, contributed to LTP (Bon & Garthwaite, 2003; Hopper & Garthwaite, 2006). The concerted roles of nNOS and eNOS helps understand the earlier observations on hippocampal LTP in mice lacking nNOSα and eNOS (Son *et al.*, 1996) although the data on nNOSα knockouts may be complicated by activity from residual nNOS splice variants (see above).

As eNOS is mostly, if not exclusively, found in endothelial cells, effects of knocking out eNOS on brain function may be interpreted as the consequence of eliminating a signal from endothelial cells to neuronal or glial cells. eNOS-knockout mice suffer disrupted synaptic plasticity not only in the hippocampus (Wilson *et al.*, 1999; see also Kantor *et al.*, 1996) but also in the cerebral cortex and striatum (Haul *et al.*, 1999; Doreulee *et al.*, 2003). In the solitary tract nucleus, eNOS regulates autonomic function and angiotensin II may influence the baroreceptor reflex here by releasing NO from endothelial cells (reviewed by Paton *et al.*, 2007). Mice lacking eNOS exhibit greatly reduced NMDA-evoked GABA release in the cerebral cortex, hippocampus and striatum, as measured using microdialysis *in vivo* (Kano *et al.*, 1998), reduced aggression (Demas *et al.*, 1999), accelerated turnover of serotonin in the frontal cortex and of dopamine in the ventral striatum (Frisch *et al.*, 2000), and decreased neurogenesis (Reif *et al.*, 2004; Chen *et al.*, 2005).

Endothelial eNOS offers a pathway for many different blood-borne agents, including hormones, to influence brain function. In some specific regions, such as the hypothalamic median eminence, eNOS may regulate the output of hormones by effecting structural adaptations (De Seranno *et al.*, 2004). Bringing eNOS into the picture also has repercussions for experimental design and interpretation because endothelial cells could be an active source of NO following application of many different experimental agents, bearing, as they can do, a long list of receptors (for acetylcholine, ADP, bradykinin, serotonin, histamine, etc) that are coupled to eNOS activation.

## Concluding remarks

This review has attempted to encapsulate the substantial progress made in recent years towards understanding the cellular and molecular mechanisms through which NO acts in the mammalian CNS, mechanisms that must ultimately explain the multifarious behavioural effects of NO evident at the whole-animal level. The broad conclusion to be drawn is that NO functions in the mammalian CNS more or less like a conventional neurotransmitter, in much the way it does in animals that evolved hundreds of millions of years ago. The main distinguishing features are the ways that it is produced and its ability to spread extremely rapidly through cell membranes away from its point of synthesis, properties that allow it to operate economically (dispensing with the need for complex storage and release devices) and in a much more versatile fashion than can be achieved with a transmitter acting only extracellularly while, at the same time, achieving a similar degree of synapse specificity. Adding to the economy is the need for so few NO molecules to do the job because the sensitivity of the NO detectors is so high: were our hypothetical NO-generating synapse (Fig. 2) going at full blast for a second, it would use up only 1000 l-arginine molecules, or roughly a quarter of the number of amino acid neurotransmitter molecules released from a single synaptic vesicle.

Most often, NO is seen to operate in concert with other transmitters rather than in a stand-alone mode; yet, by eliciting subtle alterations in the functioning of ion channels or other proteins, it can elicit effects whose consequences can be profound and sometimes very long-lasting. One of the next steps must be to establish an anatomically coherent picture of the workings of the signalling pathway at individual synapses because, unlike with conventional transmitters, there are no rules to follow and so each synapse must be treated on its own merit. A distinct shortcoming has been the lack of methods capable of measuring physiologically meaningful NO signals in real time and in subcellular dimensions. The recent development of genetically encoded fluorescent sensors for monitoring NO and cGMP with the necessary specificity, sensitivity and dynamics (Sato *et al.*, 2005; Russwurm *et al.*, 2007; Nausch *et al.*, 2008) are likely to herald progress on this issue, as well as on the subcellular regulation of nNOS and NO receptors. Further downstream, untangling the molecular mechanisms of short-term and long-term alterations occurring as a result of cGMP elevation, particularly when PKG activation is involved, will be challenging. Finally, there appears to be another, hitherto unsuspected, source of NO that may impinge importantly on CNS function, namely the capillary endothelial cells. The meaning of this line of communication is speculative but it is likely to convey a different type of message from that generated at synapses because of its global and more persistent (but low-level) nature, one that may translate peripheral signals, such as from hormones, into alterations in CNS function, or ‘prime’ neuronal responsiveness to synaptically released NO or other transmitters.

## Abbreviations

CaMK: Ca^2+^/calmodulin-dependent protein kinase
CNG channel: cyclic nucleotide-gated ion channel
CNS: central nervous system
eNOS: endothelial NOS
EPSP: excitatory postsynaptic potential
HCN channel: hyperpolarization-activated, cyclic nucleotide-modulated ion channel
Hsp: heat-shock protein
iNOS: inducible NOS
IPSC/P: inhibitory postsynaptic current/potential
LTD: long-term depression
LTP: long-term potentiation
nNOS: neuronal NOS
NO: nitric oxide
NOS: NO synthase
PDE: phosphodiesterase
PKG: cGMP-dependent protein kinase
